# Severe upper gastrointestinal hemorrhage in a renal transplant recipient: A potential complication of immunosuppressive therapy

**DOI:** 10.1177/2050313X261420025

**Published:** 2026-09-19

**Authors:** Elhassan Mahmoud, Yousra Ali, Ibrahim M. Obeidat, Souraia Mezhoud, Rafie A. Yakoob

**Affiliations:** 1 Internal Medicine Residency Program (IMRP), Division of Internal Medicine, Hamad Medical Corporation, Doha, Qatar; 2 Department of Gastroenterology, Hamad Medical Corporation, Doha, Qatar

**Keywords:** upper gastrointestinal bleed, mycophenolate mofetil, immunosuppressive therapy, kidney transplant, graft rejection

## Abstract

Upper gastrointestinal bleed (UGIB) is a significant and potentially life-threatening complication in renal transplant recipients, frequently associated with immunosuppressive therapy. We present a case of a 38-year-old male renal transplant recipient who experienced recurrent UGIB due to duodenal and gastric ulcers during the early postoperative period. His immunosuppressive regimen, initially consisting of antithymocyte globulin, steroids, tacrolimus, and mycophenolate mofetil (MMF), was adjusted multiple times in response to persistent bleeding episodes. Despite numerous endoscopic, radiological, and surgical interventions, his condition necessitated further immunosuppressive modulation, including cessation of MMF and introduction of belatacept, which ultimately stabilized his gastrointestinal condition. This case underscores the complexities of managing UGIB in renal transplant recipients under immunosuppressive therapy and highlights the need for a tailored multidisciplinary approach to optimize outcomes while preserving graft function.

## Introduction

Upper gastrointestinal bleed (UGIB) is a serious and potentially life-threatening complication in renal transplant recipients.^1^ Post-transplant patients are often at increased risk of gastrointestinal (GI) bleeding due to the immunosuppressive regimens required to prevent graft rejection.^2^ These regimens, which commonly include corticosteroids, calcineurin inhibitors, and mycophenolate mofetil (MMF), can predispose patients to mucosal injury, ulcer formation, and impaired healing.^3^ The early postoperative period, especially the first 90 days, is a critical window when immunosuppression is at its peak, increasing both infection risk and GI complications.^4^

UGIB after renal transplantation is associated with significant morbidity and mortality, particularly in patients with compromised graft functions. Known etiologies in transplant patients include erosive gastritis, peptic ulcer disease, infections (e.g. *Helicobacter pylori*), and, less commonly, opportunistic infections and neoplasms.^5^ In this context, the management of UGIB in renal transplant recipients often necessitates a multidisciplinary approach, involving endoscopic, radiological, and surgical interventions to control bleeding, alongside close monitoring and adjustment of immunosuppressive therapy to minimize adverse effects.^6^

In this case report, we present a 38-year-old male with end-stage renal disease secondary to type 1 diabetes mellitus who received a kidney transplant from a related living donor. His postoperative course was complicated by recurrent UGIB due to duodenal and gastric ulcers, which required multiple interventions. This case underscores the challenges in managing GI bleeding in the context of immunosuppressive therapy and highlights the importance of a tailored approach to immunosuppressive management to mitigate bleeding risk while ensuring graft survival.

## Case report

### Case history and examination

A 38-year-old Middle Eastern male with a history of end-stage renal disease secondary to type 1 diabetes mellitus underwent a living-donor kidney transplant from his brother. His medical history included diabetic nephropathy, diabetic retinopathy, hypertension, and hyperuricemia. He underwent a pre-kidney transplant screening, including upper and lower endoscopies, which were unremarkable with a negative Campylobacter-Like Organism test. His initial immunosuppressive regimen consisted of anti-thymocyte globulin, steroids, tacrolimus, and MMF.

The patient developed hypotension intraoperatively, which caused delayed graft function attributed to acute tubular necrosis, which was confirmed by a biopsy on postoperative day 7. The biopsy revealed severe tubular injury with focal C4d positivity and no signs of rejection. On postoperative day 10, surgical re-exploration due to persistent delayed graft function revealed a viable kidney with controllable cortical bleeding. He required intermittent dialysis for a brief period until his graft function gradually improved.

On postoperative day 15, the patient developed urosepsis due to an extended-spectrum beta-lactamase–producing *Escherichia coli*, likely secondary to an infected JJ stent, and was started on meropenem. On the same day, the patient developed significant GI bleeding, presenting with melena and fresh rectal bleeding. His hemoglobin dropped from 8 g/dL to 4.3 g/dL, and a massive transfusion protocol was initiated. The patient was kept nil per os, and intravenous pantoprazole was initiated.

### Diagnosis, investigations, and treatment

An urgent upper gastrointestinal endoscopy revealed two large visible vessels in the posterior wall of the second part of the duodenum (D2), one of which was actively oozing (Figure 1(a) and (b)). Endoscopic interventions with hemoclips and adrenaline injections failed to achieve hemostasis. A computed tomography (CT) angiogram of the abdomen subsequently demonstrated active contrast extravasation within the third part of the duodenum, consistent with ongoing bleeding (Figure 2(a) and (b)). Embolization of the gastroduodenal artery by interventional radiology was unsuccessful, necessitating exploratory laparotomy with duodenotomy and suture ligation of bleeding vessels in the D2/D3 region. Following surgery, his immunosuppressive regimen was adjusted to intravenous steroids, tacrolimus, and MMF. His urine output gradually improved, and he no longer required dialysis.

**Figure 1.**
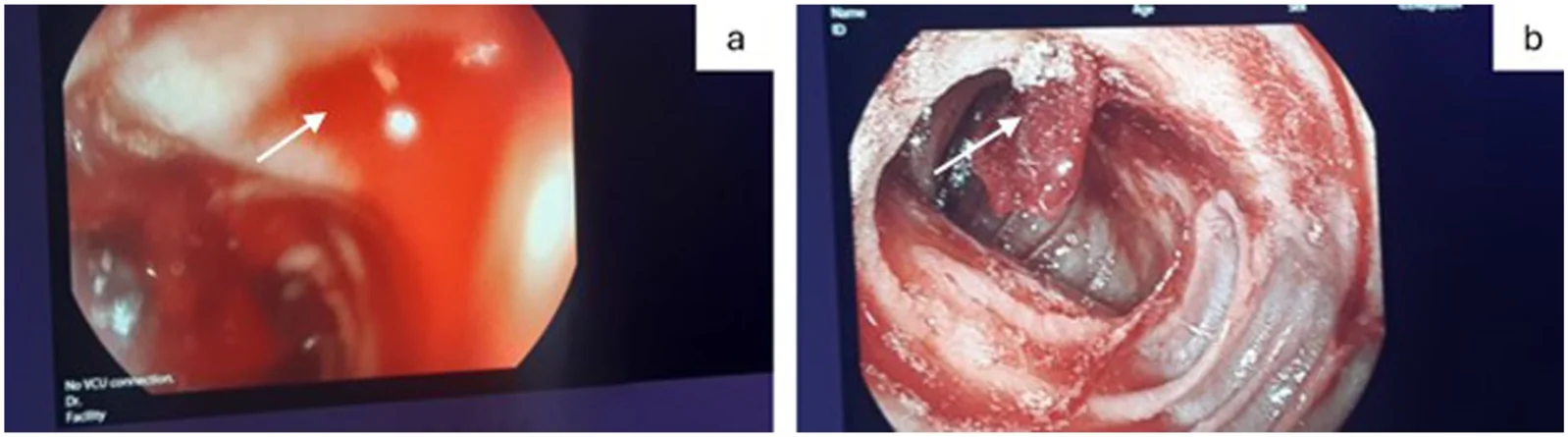
Endoscopic findings in the duodenum on the first upper GI endoscopy on postoperative day 15. (a) Actively oozing vessel located at the posterior wall of the second part of the duodenum (Forrest 1b). (b) A visible vessel with an overlying adherent clot located at second part of the duodenum (Forrest 2a). Arrows indicate the location of the lesions. GI: Gastrointestinal.

**Figure 2.**
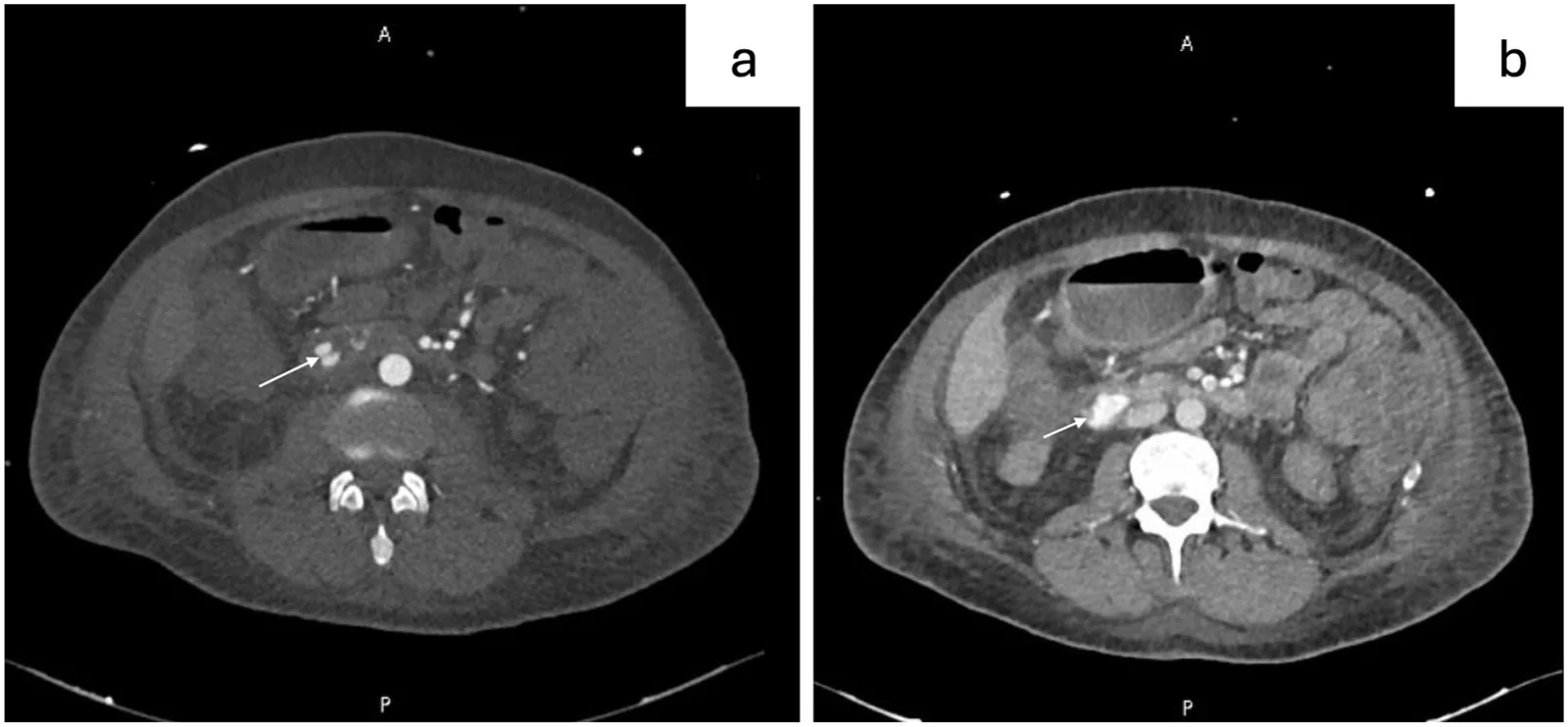
CT angiogram of the abdomen demonstrating active duodenal bleeding (a) Arterial phase: Active contrast extravasation within the third part of the duodenum, indicated by the arrow (b) Venous phase: Pooling of contrast in the same region, marked by the arrow. CT: Computed tomography.

On postoperative day 25, the patient developed melena. An upper GI endoscopy revealed multiple new ulcers in the stomach and duodenum, including a visible vessel in the duodenal bulb, which was treated with hemoclips, adrenaline injections, and gold probe cautery. Another ulcer was noted in D3 along the lateral wall with no underlying vessel. Intravenous steroids were also discontinued, and he was maintained on MMF and tacrolimus. Despite these interventions, the patient continued to experience drops in hemoglobin and recurrent melena. Repeated upper endoscopy on postoperative day 27 showed circumferential ulceration of the distal esophagus and further ulcers in the duodenum with active oozing, requiring additional hemoclips, adrenaline injections, and Purostat gel application. The patient’s condition deteriorated, requiring intubation for respiratory distress.

On postoperative day 28, a CT angiogram was done, which demonstrated a large periduodenal hematoma without active bleeding. On postoperative day 29, angiography revealed contrast extravasation from a distal branch of the inferior pancreaticoduodenal arcade at the gastroduodenal artery junction. Embolization was attempted with 300–500 µm Embosphere particles under fluoroscopic guidance, resulting in sluggish flow to the target vessel and sufficient collateral circulation. Subsequent upper endoscopy on postoperative day 29 showed severe esophagitis with friable mucosa, clotted blood in the stomach, and several ulcers in the stomach (Forrest 2a) and duodenum at the D1/D2 junction (Forrest 1b). Endoscopic intervention included adrenaline injections and Endoclip for the ulcers. Biopsy of the duodenum revealed peptic duodenitis with mild villous blunting, and Cytomegalovirus (CMV) was negative.

The patient was managed with total parenteral nutrition, and continuous renal replacement therapy was initiated for volume overload. MMF was stopped due to the persistent GI bleeding, and the immunosuppressive regimen was shifted to intravenous tacrolimus, oral azathioprine, and intravenous belatacept every 2 weeks.

### Outcome and follow-up

The patient’s condition gradually stabilized, as he did not develop further GI bleeding, and his graft function improved. He was extubated, oral feeding was gradually resumed, sucralfate was added, and his renal function improved without further need for dialysis. The patient’s final session of continuous renal replacement was on postoperative day 40. Once oral feeding was fully tolerated, intravenous belatacept was discontinued. The patient was discharged on postoperative day 53 on tacrolimus and azathioprine with a stable creatinine level of 95 µmol/L and no new episodes of GI bleeding.

## Discussion

We presented a case of significant UGIB due to duodenal and gastric ulcers in the early postoperative period following renal transplant. Despite multiple endoscopic, radiological, and surgical interventions, the patient’s bleeding persisted, suggesting a potential role of vascular fragility or mucosal damage exacerbated by immunosuppressive therapy. UGIB post-renal transplant is well reported as a frequent complication that is associated with high morbidity and mortality rates,^7^ especially in the setting of allograft dysfunction.^8^ The incidence of post-kidney transplant GI bleed is around 5%–12%, occurring during the first post-kidney transplant year when the doses of immunosuppressants are highest.^9^

The risk is increased with age, a deceased donor rather than living donor, and active CMV infection. The common sources of GI bleeding in transplant patients include duodenal ulcers, erosive gastritis,^6^ *H. pylori* infection, and bleeding from solid tumors.^9^ The risk of GI bleeding is high in the first 90 days post-transplant. Similarly, procedure-related complications, maximal doses of steroids use, immunosuppressive therapy, and possibilities for renal replacement therapy are high during the same period,^10^ which further increases the bleeding risk. However, selecting healthier transplant recipients reduces the overall bleeding risk, as they typically require less exposure to medications such as heparin during hemodialysis and are less likely to experience uremia-related platelets dysfunction and vascular access complications.^9^ Moreover, another possible cause of GI bleeding in post-renal transplant patients includes inflammatory ulcers resembling those in inflammatory bowel disease, which may respond to a single dose of infliximab.^11^ These ulcers develop secondary to the derangement of the immune system, which is further aggravated by high doses of immunosuppressive treatment administered during the postoperative period. However, since the patient improved following adjustment of immunosuppressive therapy, treatment with infliximab was deemed unnecessary.

In recent years, several immunosuppressive agents have been associated with GI bleeding in transplant patients. Our case provides further evidence that agents such as MMF may contribute to GI bleeding, particularly in patients without prior GI risk factors. Our patient, who was negative for *H. pylori* and CMV, had no previous history of GI bleeding, and initially received a regimen including MMF, experienced recurrent, severe upper GI bleeds post-transplant. Despite multiple endoscopic and interventional treatments, bleeding persisted until MMF was discontinued, at which point the bleeding episodes resolved and his condition stabilized. This clinical improvement following immunosuppressive adjustment supports a potential role of MMF in exacerbating GI mucosal vulnerability.

MMF-induced gastrointestinal toxicity is thought to result from inhibition of inosine monophosphate dehydrogenase, a key enzyme in de novo purine synthesis.^12^ Because enterocytes rely heavily on this pathway, MMF can impair mucosal regeneration, induce enterocyte apoptosis, and disrupt epithelial barrier integrity, predisposing to ulceration and bleeding. In contrast, belatacept exerts its immunosuppressive effect through selective T-cell co-stimulation blockade and has a substantially lower rate of gastrointestinal adverse events, making it a suitable alternative in patients with MMF-related mucosal injury.^13^

Several alternative etiologies of gastrointestinal bleeding (GIB) in the early post-transplant period were considered, including corticosteroid-induced ulceration, stress-related mucosal disease, and uremia-related platelet dysfunction in the setting of delayed graft function. However, stable coagulation parameters, negative CMV biopsy, and the presence of recurrent focal ulcers made these causes less likely. The resolution of bleeding only after discontinuation of MMF further supported a drug-related mucosal injury rather than a secondary process.

Our findings suggest that multiple factors, including surgical stress, corticosteroid administration, and MMF therapy, may contribute to GIB following kidney transplantation. In our patient, specifically, potential triggering factors included perioperative use of corticosteroids administered when graft rejection was initially suspected, MMF therapy, and significant vascular challenges complicating attempts. CMV infection was unlikely, given negative biopsy findings. These observations align with previous reports linking MMF to GI complications. For example, Prueksapanich et al.^11^ reported a kidney transplant patient with severe duodenal ulcers who, like our patient, was *H. pylori*-negative and experienced resolution of GI symptoms only after MMF was stopped and infliximab was initiated. Similarly, in a liver transplant case by Heo et al.,^14^ a patient on MMF developed GI bleeding, and bleeding control was achieved through dose adjustments of immunosuppressive therapy. Abbass et al.^15^ also observed a heart transplant patient who, following discontinuation of MMF, showed no recurrence of GIB over a 10-week follow-up. These cases show a pattern where MMF, despite its efficacy in preventing rejection, may increase the risk of GI bleeding, even in patients without common risk factors. It further underscores the importance of immunosuppressive modification when managing GI bleeding in transplant recipients.

Although MMF-associated GIB has been described in the literature, the clinical course in our patient differed in several meaningful ways. Previously reported cases generally involved later-onset bleeding, more localized ulceration, or successful control with a single interventional modality or immunosuppressive adjustment. In contrast, our patient experienced very early, multifocal ulceration involving the esophagus, stomach, and duodenum, together with refractory hemorrhage requiring multiple endoscopic sessions, unsuccessful embolization attempts, and surgical duodenotomy before bleeding could be controlled. Sustained improvement occurred only after complete discontinuation of MMF and conversion to a belatacept-based regimen. Table 1 summarizes key differences between prior reports and the present case.

**Table 1.** Comparison of previously reported MMF-associated gastrointestinal bleeding cases with the present case.

Variable	Prueksapanich et al.^11^	Heo et al.^14^	Abbass et al.^15^	Present case
Age / Sex	50 / F	65 / M	56 / M	38 / M
Transplant Type	Kidney	Liver	Heart	Kidney
Bleeding onset	1 week post-transplant	3 weeks post-transplant	5 months post-transplant	2 weeks post-transplant
Initial immunosuppressive therapy	ATG, steroids, tacrolimus, MMF	Tacrolimus, steroids, MMF	MMF, tacrolimus, prednisone	ATG, steroids, tacrolimus, MMF
*H. pylori* status	Positive	Negative	Negative	Negative
CMV status	Negative	Not tested	Negative	Negative
Adjustment to immunosuppression	Stopped steroids and MMF	Reduced all doses; stopped MMF; tacrolimus → cyclosporine	Stopped MMF → azathioprine	Stopped MMF → belatacept + azathioprine + IV tacrolimus
Therapeutic interventions	Endoscopy; angiographic embolization; infliximab	Endoscopy; embolization; segmental resection; thalidomide	PPI + sucralfate	Multiple endoscopies; failed embolization; surgical duodenotomy; CRRT; ICU care
Outcome	No recurrence at 3 months	No recurrence; stable graft	Symptom resolution at 10 weeks	No recurrence; stable graft at discharge (POD 53)

ATG: Antithymocyte globulin; MMF: Mycophenolate mofetil; CMV: Cytomegalovirus; PPI: Proton pump inhibitor; CRRT: continuous renal replacement therapy; ICU: Intensive care unit; POD: Postoperative day.

Furthermore, the management of GI bleeding in post-transplant patients is challenging and requires a multidisciplinary approach, as evidenced by studies reporting the need for repeated endoscopic and radiologic interventions to control bleeding. For instance, Prueksapanich et al.^11^ presented a kidney transplant patient with recurrent duodenal ulcers requiring multiple endoscopic procedures before MMF discontinuation, who eventually controlled the bleeding. Similarly, Heo et al.^14^ reported a liver transplant patient who underwent angiographic embolization, segmental bowel resection, and started thalidomide before achieving hemostasis. These cases, including our case, showcase the challenges and complexity of managing GI bleeding in this population, where endoscopic, surgical, and medical interventions are often employed simultaneously to address persistent bleeding.

Despite growing recognition of immunosuppression-related gastrointestinal injury, several aspects of management remain unresolved. There are no standardized guidelines on how to modify immunosuppressive therapy during active, life-threatening bleeding, and decisions are often individualized based on institutional or clinical experience. Distinguishing MMF-induced mucosal injury from alternative causes, such as steroid-associated ulceration, stress-related mucosal disease, uremia-related platelet dysfunction, or ischemic injury, is difficult, as no validated endoscopic or histologic criteria reliably differentiate these entities. In addition, the optimal choice and timing of alternative agents, such as belatacept or azathioprine, are not well defined in the early postoperative period when rejection risk is highest. These gaps emphasize the need for consensus recommendations and prospective studies to guide immunosuppressive adjustment in transplant recipients presenting with severe or recurrent GIB.

A strength of this report is the detailed documentation of sequential endoscopic, radiologic, surgical, and pharmacologic interventions, as well as the clear temporal relationship between immunosuppressive modification and clinical improvement. However, the case is limited by the emergent nature of the interventions, which restricted the availability of high-resolution endoscopic imaging, and by the inherent limitations of a single-patient observation, which may affect generalizability. Despite these limitations, the case provides meaningful insight into the potential gastrointestinal toxicity of MMF and the role of alternative immunosuppressive strategies.

## Conclusion

In conclusion, in our experience, the case of our patient demonstrates the importance of considering immunosuppressive agents as the potential culprit to GI bleeding in transplant recipients, even in the absence of traditional risk factors. We recommend the identification of patients at high risk for GIB, such as those with a history of GIB, peptic ulcer disease, gastroesophageal reflux disease, or *H. pylori* infection, to facilitate early preventive measures. Rapid withdrawal of corticosteroids when clinically appropriate, along with prophylaxis using proton pump inhibitors, may help reduce bleeding risk. In addition, we recommend close monitoring of gastrointestinal symptoms in patients receiving MMF, and prompt immunosuppressive adjustments at the onset of GIB. Ultimately, a collaborative, multidisciplinary approach tailored to individual patients is critical to effectively balance the necessity of immunosuppression against its potential gastrointestinal toxicity.

## Data Availability

The data that support the findings of this study are available from the corresponding author upon reasonable request.*
